# Supplementary material for: Status of the stateless population in Thailand: How does stigma matter in their life?
Source: PLoS One. 2022 Mar 14;17(3):e0264959. doi: 10.1371/journal.pone.0264959 (PMC8920272; doi:10.1371/journal.pone.0264959)
Supplement: S1 Appendix — (DOCX) [file pone.0264959.s001.docx]

**Questions guide**

1) What do you think about being a member of a ‘stateless population’?”

2) Have you experienced any events that have made you feel uncomfortable when you have contacted people or health care providers?

3) Could you please tell me about stigma-related situations that you face in your daily life?

4) How do these situations impact you and your life?

5) How do you cope with these situations?

6) What other expectations do you have about such a stigma?
