# Supplementary material for: Status of the stateless population in Thailand: How does stigma matter in their life?
Source: PLoS One. 2022 Mar 14;17(3):e0264959. doi: 10.1371/journal.pone.0264959 (PMC8920272; doi:10.1371/journal.pone.0264959)
Supplement: S2 Appendix — (DOCX) [file pone.0264959.s002.docx]

A: Mrs….., the following questions will be the earlier mentioned ones I asked you. Your information will not be sent to the hospital or Public Health Center. Instead, it will be collected and discussed in my own paper.

B: Ahh, I got it.

A: And this interview will be held as a project.

B: I see

A: Let’s start…How old are you now?

B: My age might be about 54 years old. I’m not sure about it.

A: What is your exact age? Did you have late birth certificate?

B: I don’t have any certificate. I am not Thai.

A: What do you mean by “I am not Thai”?

B: I don’t have Thai Identification card. This is show that I am not Thai even though I really want to be Thai and really want to get ID card from the government.

A: I have heard that you can request for Thai ID card.

B: Yes, I have asked the head of my village many times

A: What’s your religion?

B: I am Christian.

A: How many people in your house?

B: There are 4 people in my house, husband, daughter, son and myself.

A: All of your family member are stateless people?

B: No, my son and my daughter are Thai. They got the Thai National Identification card, only me and my husband are stateless people.

A: Why?

B: Because my children, they born here. I and my husband were born in Myanmar and we migrated to Chiang Rai 30 years ago. We don’t have any certificate from the Thai government. But my children have it both.

A: This is mean your children have got the Thai ID card since they were born in Thailand?

B: Yes, I gave birth at the hospital and my children are Thai automatically. If I was born in Thailand, I think I would get Thai Id card without any reason.

A: Where are your two young children?

B: My oldest child is in Bangkok. The second child is in Chiang Rai.

A: They already have works.

B: They are studying. They are both studying and working. The youngest child now doesn’t work and only studies.

A: The youngest is in Chiang Rai.

B: He lives there for 11 years

A: Where does he study?

B: Chiang Rai Vocational Education College. He studies at Chiang Rai Vocational Education College.

A: Ahh. Is it good? Do you have enough money for him? Does it cost much money?

B: So much money

A: You pay for your child’s education.

B: He wants to further study. I can’t support him more. It is good for him to earn money by himself in order to pay for study. My child told he would do it. He won’t get money from me.

A: What is his job?

B: I don’t know. He doesn’t do it. He doesn’t work now. He would look for a job when starting study. He is now on holidays.

A: Does he look for scholarship?

B: He got scholarship from H.R.H Princess Maha Chakri Sirindhorn.

A: He received H.R.H Princess Maha Chakri Sirindhorn’s scholarship. He must be intelligent.

B: Yes. H.R.H Princess Maha Chakri Sirindhorn’s scholarship. So, he can study further.

A: Did you attend the school?

B: No, I didn’t. Nobody in my village attended the school and the school is very far from our village. Actually, we didn’t have right to go to the school due to we are the stateless people.

A: Where did you learn Thai from?

B: We communicate with Thai, sometimes go to town center for buying goods in the biggest market so we have to learn Thai.

A: What’s your occupation?

B: I am a farmer but the land is not mine. I rent from the landlord so just plant for living. I mean not for sale the rice.

A: How much you earn each month?

B: I am not sure, it’s around 1,000-2,000 baht.

A: Is this amount of money enough for your living cost?

B: Yes, living in this village, you don’t need much money because I can find food in the forest. I can get the fish from the river and I plant the rice for eating. But if I go to the town center, I feel I am very poor because everything is very expensive. I have to pay for the transportation or hire a car from people in my village for example.

A: What do you think about being a member of a ‘stateless population?

B: I don’t know what to say. It’s inside my heart. I’m telling you. I feel like I am different from other people who have got Thai ID card. I feel I don’t have the right to do things even the right for my health to get the health care services.

A: Did the staffs speak polite to you when you go to the hospital or get the health care services?

B: In the past about 10-15 years ago no, they didn’t. They made me felt shy and did not have confidence because I could not speak Thai.

A: Do you mean around 10-15 years ago; you could not speak Thai and the staffs look down on you and you can feel it because they did not speak to you politely?

B: Exactly, I felt that they look down on me by speaking infuriate to me. I think they did not know that I understand Thai but I could not speak. I heard someone there gossip me too.

A: What did they gossip about you?

B: I heard that they talked to each other that I am hill tribe and I am not a Thai citizen and complained that they were bored when to deal with stateless people like me.

A: And what about nowadays?

B: All staff are good and talk to me politely.

A: How do you feel in terms of speaking politely to you?

B: I feel much better than that time. At least I am a human and they speak to me like other Thai people. I mean their voice, polite and good service.

A: Please tell me about your feeling when you go to go to get the health care services.

B: I don’t have any power to request anything from the hospital because I am the stateless people. I don’t have ID card. When go to the hospital, the staffs always ask for my ID card and eventually, they put the red pen in a paper and I know that means I have no right to get the free chart of the services. It’s always like this all my life.

A: Have you ever asked someone to do or request this for you?

B: No, never. I have been facing this situation to all my life and I know it cannot be changed anything.

A: Have you experienced any events that have made you feel uncomfortable when you have contacted people or health care providers?

B: I had a stomachache a month ago, and I went to see a doctor in a hospital. He prescribed some medicines to me, but they made me feel only a little better. I truly did not know what I should do next. I hoped I would be referred to a larger hospital in the city. However, I had no idea because I have no Thai ID. I do not have any right to ask for help from him. It is like no one can hear my voice.

A: Could you please tell me about stigma-related situations that you face in your daily life?

B: One day I went to the town center to buy things for the ceremony in the village. The seller did not talk to me softly. I felt he look down on me. I asked him to see the cloth and he acted like he does not want to sell it. I think because I don’t speak Thai clearly and I have the different accent from general Thai people.

A: How do these situations impact you and your life?

B: I have to face these situations with all my life. I just accept and stay the way I am. It can not be help. I born to be like this. I hope the next life I will be born in Thai territory. And I will have Thai ID card automatically.

A: How do you cope with these situations?

B: As I told you, nothing I can do. I just accept the way they have talked to me. And I try to avoid to go to the town center on my own. I need someone or friend in my village to come with me. In case I cannot understand Thai, these people can help me.

A: What other expectations do you have about such a stigma?”

B: I just want everyone to accept me as a human being. I am a human like them and I have feeling. Don’t laugh at me. Also, I really want the Thai ID card.

A: Why does the ID card necessary for you?

B: It’s very important, without ID card I have no rights as same as Thai or the people in the village who have it. I have to pay when I go to see the doctor. I have to pay everything while the people who have ID card does not have to.
