# Supplementary material for: Status of the stateless population in Thailand: How does stigma matter in their life?
Source: PLoS One. 2022 Mar 14;17(3):e0264959. doi: 10.1371/journal.pone.0264959 (PMC8920272; doi:10.1371/journal.pone.0264959)
Supplement: Attachment — Submitted filename: Response to reveiwer commentsEE.docx [file pone.0264959.s003.docx]

**Response to reviewers’ comments**

Dear Plosone,

Thank you very much for given us the opportunity to revise the manuscript. We have carefully checked, cleared, and responded to all points raised by you and reviewers.

Moreover, we have made a bit change on the title from “Status of the stateless population in Thailand: how does stigma matter in their life?” to “Status of the stateless population in Thailand: how does stigma matter in their life ?” which is reported in the cover letter to you as well.

We do hope that you happy in this version, and look forward to hearing from you.

Regards,

TK

PONE-D-21-27010

Status of the stateless population in Thailand: how does stigma matter in their life?

PLOS ONE


Journal Requirements:

When submitting your revision, we need you to address these additional requirements.

1. Please ensure that your manuscript meets PLOS ONE's style requirements, including those for file naming. The PLOS ONE style templates can be found at 
<https://journals.plos.org/plosone/s/file?id=wjVg/PLOSOne_formatting_sample_main_body.pdf> and 
<https://journals.plos.org/plosone/s/file?id=ba62/PLOSOne_formatting_sample_title_authors_affiliations.pdf>

: Thank you, we checked and followed the journal guidelines.

2. We note that the grant information you provided in the ‘Funding Information’ and ‘Financial Disclosure’ sections do not match.

When you resubmit, please ensure that you provide the correct grant numbers for the awards you received for your study in the ‘Funding Information’ section.

: Thank you. This problem is now revised.

3. Thank you for stating the following in the Acknowledgments/ Funding Section of your manuscript:

This study was supported by a grant from the Center of Excellence for Hill Tribe Health Research, Mae Fa Luang Thailand. The funder had no role in the design of the study, collection, analysis, and interpretation of the data and writing of the manuscript.

Please note that funding information should not appear in the Acknowledgments section or other areas of your manuscript. We will only publish funding information present in the Funding Statement section of the online submission form.

Please remove any funding-related text from the manuscript and let us know how you would like to update your Funding Statement. Currently, your Funding Statement reads as follows:

The funders had no role in study design, data collection and analysis, decision to publish, or preparation of the manuscript.

Please include your amended statements within your cover letter; we will change the online submission form on your behalf.

: Thank you. This information is now revised.

: Please kindly insert the following statement in the funding section:

“**Funding**

This study was supported by a grant from the Center of Excellence for Hill Tribe Health Research, Mae Fa Luang Thailand (No. 01-2021). The funder had no role in the design of the study; collection, analysis, and interpretation of the data; or writing of the manuscript.”

4. Please include captions for your Supporting Information files at the end of your manuscript, and update any in-text citations to match accordingly. Please see our Supporting Information guidelines for more information: <http://journals.plos.org/plosone/s/supporting-information>.

: Thank you. These files are now revised and updated.

[Note: HTML markup is below. Please do not edit.]

Reviewers' comments:

5. Review Comments to the Author

Please use the space provided to explain your answers to the questions above. You may also include additional comments for the author, including concerns about dual publication, research ethics, or publication ethics. (Please upload your review as an attachment if it exceeds 20,000 characters)

Reviewer #1:

1) The paper is interesting and reflects the significant issue that minorities in Thailand are facing.The manuscript is well structured. However, there are some grammatical errors (for example, tenses) and word choices (for example, a mole instead of a spy).

: This manuscript was revised again by American Journal Experts (AJE), reference No 39DA-241D-833B-CF5B-531B

2) The authors need to work more on literature reviews. More discussions on the differences between stigma, prejudice, and discrimination should be added. More reviews on types of stigma: public stigma, self-stigma, & institutional stigma could be a good addition.

: Thank you for the comment. We added the relevant information on page 3, lines 7-21 with references no 15-19.

3) The methodology section is well structured and clear.

: Thank you.

4) Some contents in the result section are not matched with headers in the result section, for example, in sections C, D, & E. The examples related to the Thai language may have a separate section.

: Thank you for this important comment.

: As a team, we carefully discussed this issue and realized that in Parts C and D, two themes were extracted from the analysis: being unable to qualify for health care services and being ranked below other hill tribe people who do hold Thai IDs. We revised all the details of the supported content so it relates to the theme. Meanwhile in Section E, P#9 said she could not get a good job because she did not attend a school when she was young. She also said she could not speak Thai, which was one reason she was not recruited into a good job. We decided to retain the sequence of her story in the manuscript’s contents because we want to ensure that all readers understand the context of the stateless population in Thailand.

: With this important comment, we revised all the contents in the manuscript to ensure that all the information and themes are consistent.

: Thank you very much for the comment and we hope our revisions are clear.

5) It is unclear on the interpretation of “life is nothing.” Is it about meaningless or invisible?

: After carefully reviewing Section E (“Life is nothing”), we completely agree with you that the message lacks interpretation. Thank you very much for the important comment.

: We reviewed all the information and interpreted it. Please see page 11, lines 12-14.

6) In the conclusion section, the authors may need to suggest more substantial implications or policy recommendations instead of summarizing the findings again.

: Thank you for the comment. We improved this section.

Reviewer #2:

1) The authors aimed to examine the experience of Thailand's stateless population and how they deal with stigma. The manuscript is a technically sound piece of scientific research that includes data to back up the conclusions. There is a concise statement as well as ethical considerations.

: Thank you very much.

2) However, there is a lack of theoretical support/discussion. An additional topic of debate might be the concept of stigma in marginalized communities, i.e., Furthermore, the analytical component is demonstrated without a comprehensive literature study or theoretical support/discussion.

: Thank you for the comment.

: Essentially, we reviewed many studies and examined many concentrated conceptual frameworks before undertaking this project. Ultimately, we used the framework integrating normative influence on stigma (FINIS) for the study [15], which turned out to the best fit.

3) The analytical focus is solely on Thailand, with only limited implications for other empirical investigations. The author provides no evidence of demographic differences or differing educational backgrounds, which may have an impact on the type/degree of stigma. In many cases, the transcripts were simply inserted without further examination.

: Thank you for the important comment. This project originated from the stigma experiences among the stateless population in Thailand. During the analysis, we thought carefully about including individual information in the manuscript quotations. However, we realized that inserting increasing amounts of information into the manuscript might violate the ethics of a good human-subject research project. Therefore, we listed the group’s demographics, including education, in Table 1.

: Most of the stateless population in Thailand cannot access education (66.7% had no education and 23.5% attended primary school only), and the stigma impacts may not differ according to their educational level. We hope our explanation is clear.

4) Please clearly specify the interviewee selection criteria.

: The interviewee selection criteria were a) being part of the stateless population, defined as having no Thai ID, and b) experiencing any form of stigma (page 4, lines 19-21).

5) Furthermore, because the definition of a stateless person is "who is not considered as a national by any state under the operation of its law" (The 1954 Convention relating to the Status of Stateless Persons), Simply described, a stateless person is someone who does not have nationality from any country. Thereby, the analytical and conclusion sections, as well as recommendations should be done with caution to avoid recommendations with a tautology-related perspective.

: Thank you so much for such an important comment.

: The first paragraph of the Conclusion was focused on granting Thai IDs because the context of the related laws has changed over time and by case. Therefore, the 1954 Convention relating to the Status of Stateless Persons clearly states the definition of stateless persons, but the new Constitution of Thailand includes much evidence as new criteria to be used for granting IDs. These include making a great contribution to Thai society in any respect, being a person devoted to Thailand through sports, etc. Moreover, the law has been revised to adapt to the new situation of Thai society. Therefore, we included this content in the first paragraph of the Conclusion. We hope that our reasoning is clear.

TK

Assistant Professor Dr. Tawatchai Apidechkul

Dean, School of Health Science, MFU

Director, Center of Excellence of the Hill Tribe Health Research, WHO-CC

Former Hubert H Humphrey Fellow (2013-2014), Emory University

Global Health Delivery Intensive (Harvard School of Public Health)
