# Supplementary material for: Status of the stateless population in Thailand: How does stigma matter in their life?
Source: PLoS One. 2022 Mar 14;17(3):e0264959. doi: 10.1371/journal.pone.0264959 (PMC8920272; doi:10.1371/journal.pone.0264959)
Supplement: Attachment — Submitted filename: Response to reviewer comments.docx [file pone.0264959.s004.docx]

**Response to reviewer comments**

Journal Requirements:

Please review your reference list to ensure that it is complete and correct. If you have cited papers that have been retracted, please include the rationale for doing so in the manuscript text, or remove these references and replace them with relevant current references. Any changes to the reference list should be mentioned in the rebuttal letter that accompanies your revised manuscript. If you need to cite a retracted article, indicate the article’s retracted status in the References list and also include a citation and full reference for the retraction notice.

: Thank you, we have double check all references. Reference No.7, 9, 16, 17, 18, 26,33, and 34 are revised and completed.


**Reviewers' comments:**

**Comments to the Author**

1. If the authors have adequately addressed your comments raised in a previous round of review and you feel that this manuscript is now acceptable for publication, you may indicate that here to bypass the “Comments to the Author” section, enter your conflict of interest statement in the “Confidential to Editor” section, and submit your "Accept" recommendation.

Reviewer #1: All comments have been addressed

Reviewer #2: All comments have been addressed

2. Is the manuscript technically sound, and do the data support the conclusions?

The manuscript must describe a technically sound piece of scientific research with data that supports the conclusions. Experiments must have been conducted rigorously, with appropriate controls, replication, and sample sizes. The conclusions must be drawn appropriately based on the data presented.

Reviewer #1: Yes

Reviewer #2: Partly

3. Has the statistical analysis been performed appropriately and rigorously?

Reviewer #1: N/A

Reviewer #2: Yes

4. Have the authors made all data underlying the findings in their manuscript fully available?

The [PLOS Data policy](http://www.plosone.org/static/policies.action#sharing) requires authors to make all data underlying the findings described in their manuscript fully available without restriction, with rare exception (please refer to the Data Availability Statement in the manuscript PDF file). The data should be provided as part of the manuscript or its supporting information, or deposited to a public repository. For example, in addition to summary statistics, the data points behind means, medians and variance measures should be available. If there are restrictions on publicly sharing data—e.g. participant privacy or use of data from a third party—those must be specified.

Reviewer #1: Yes

Reviewer #2: Yes

5. Is the manuscript presented in an intelligible fashion and written in standard English?

PLOS ONE does not copyedit accepted manuscripts, so the language in submitted articles must be clear, correct, and unambiguous. Any typographical or grammatical errors should be corrected at revision, so please note any specific errors here.

Reviewer #1: Yes

Reviewer #2: Yes

6. Review Comments to the Author

Please use the space provided to explain your answers to the questions above. You may also include additional comments for the author, including concerns about dual publication, research ethics, or publication ethics. (Please upload your review as an attachment if it exceeds 20,000 characters)

Reviewer #1: The authors addressed and responded to all of my comments and suggestions. However, I have some comments on their responses.

The name of Section E can be “No better options in life” instead of “Life is nothing.” “No better options in life” may represent the stories in this section clearer. The term “life is nothing” is unclear.

: Thank you, we agreed with you and have changed accordingly, please see page 11, line 4.

In addition, please be careful on the lettering of section “B. Perceptions of stigma.” Should they all be “d), e)”? Another option is using numbers. It may be easier to structure the sections.

: To make sure that the sequence is not difficult for readers, we have revised by using Greek alphabet (I, II...) to the main theme and use English in sub-theme. Thank you

In the conclusion section, the authors suggest that the problems may be alleviated by providing Thai IDs to hill tribe people. They also suggest the promotion of nonstigma behaviors and services toward anyone. In many countries, there are laws on discrimination in healthcare, employment, and education. The authors may explore more on the discrimination laws and regulations.

: Thank you for great comment here, we have added information on creating and implementing relevant laws on discrimination protection in access healthcare services in this section, please see in page 18, lines 27-29.

Like my comment on the subsection “e)”, the term “life is nothing” is unclear. I would suggest that the term “No better options in life” should also be used in the discussion and conclusion.

: We agreed with you and revised.

Reviewer #2: (No Response)

7. PLOS authors have the option to publish the peer review history of their article ([what does this mean?](https://journals.plos.org/plosone/s/editorial-and-peer-review-process#loc-peer-review-history)). If published, this will include your full peer review and any attached files.


If you choose “no”, your identity will remain anonymous but your review may still be made public.


**Do you want your identity to be public for this peer review?** For information about this choice, including consent withdrawal, please see our [Privacy Policy](https://www.plos.org/privacy-policy).

Reviewer #1: No

Reviewer #2: No
